# Supplementary material for: The Male-Biased Expression of miR-2954 Is Involved in the Male Pathway of Chicken Sex Differentiation
Source: Cells. 2022 Dec 20;12(1):4. doi: 10.3390/cells12010004 (PMC9818168; doi:10.3390/cells12010004)
Supplement: Supplementary file 1 [file cells-12-00004-s001.zip › cells-2030631-supplementary.pdf]

Table S1. Differentially expressed miRNAs information.

miRNAs	location	Stage 25			Stage 45			Stage 55		
		F25.tpm	M25.tpm	LogFC (F:M)	F45.tpm	M45.tpm	LogFC (F:M)	F55.tpm	M55.tpm	LogFC (F:M)
gga-miR-7b	gb|JH376210.1|	127.52	2.19	6.13 (**)	40.36	32.58	0.41	742.66	0.00	10.48 (**)
gga-miR-122-5p	chrZ, gb|JH375727.1	2395.41	5082.06	-0.81	8926.24	10603.86	-0.15	490.66	1759.94	-1.95 (**)
gga-miR-7	chrZ,chr10,chr28	216.66	369.66	-0.50	229.78	340.60	-0.47	916.18	3454.35	-2.02 (**)
gga-miR-1456-5p	chrZ	978.86	2128.67	-0.85	550.50	711.33	-0.27	215.16	638.68	-1.68 (**)
gga-miR-1459	chrZ	7.01	26.45	-1.65 (**)	1.56	1.36	0.30	0.00	0.00	NA
gga-miR-2131-5p	chrZ	143.22	417.58	-1.27 (**)	92.25	148.81	-0.59	69.94	123.33	-0.92
gga-miR-2954	chrZ	2764.45	27522.04	-3.04 (**)	2332.87	10317.46	-2.04 (**)	1382.82	5872.64	-2.19 (**)
gga-miR-31-5p	chrZ	5046.44	18193.92	-1.58 (**)	676.76	1024.46	-0.50	1734.66	9279.92	-2.52 (**)
gga-miR-214	chr8	179.46	101.15	1.10 (**)	891.90	1108.38	-0.21	868.67	1177.86	-0.54
gga-miR-202-3p	Chr6	2.71	4.13	-0.34	3.80	3.81	0.10	225.31	779.88	-1.90 (**)
gga-miR-1811	Chr4	22.22	71.25	-1.41 (**)	0.37	0.27	0.56	0.00	1.19	NA
gga-miR-133b	Chr3	106.50	51.88	1.31 (**)	71.69	47.13	0.71	3.74	2.38	0.54
gga-miR-206	chr3	39108.12	30202.29	0.64	21219.39	20141.03	0.18	5263.78	16971.49	-1.79 (**)
gga-miR-456-3p	chr3	9885.14	10214.99	0.22	4807.88	5825.52	-0.18	479.45	959.81	-1.11 (**)
novel_771	chr3	6.29	10.61	-0.48	3.24	2.99	0.21	22.42	53.02	-1.35 (**)
gga-miR-9-3p	chr28,chrZ	214.43	106.63	1.28 (**)	5148.54	7972.70	-0.53	1593.18	2700.09	-0.87
gga-miR-196-5p	chr27,chr2,JH375593.1	759.49	2197.14	-1.26 (**)	750.76	740.30	0.12	36767.99	44819.01	-0.39
gga-miR-2188-5p	chr22	64.44	85.23	-0.13	82.03	33.87	1.38 (**)	52.32	15.49	1.65 (**)
gga-miR-	chr20	62404.2	38863.86	0.95	24697.24	24348.28	0.12	47.52	101.28	-1.20 (**)

499-5p		5								
gga-miR-124a-3p	chr2	7575.99	7405.29	0.30	6866.06	19411.00	-1.40 (**)	2.14	1.79	0.15
gga-miR-124c-5p	chr2	455.86	291.92	0.91	443.12	1235.90	-1.38 (**)	1.60	1.79	-0.26
gga-miR-153-3p	chr2	89.45	121.45	-0.17	29.46	71.55	-1.18 (**)	10.14	13.11	-0.48
gga-miR-1759-5p	chr2	5.81	5.56	0.34	14.89	11.15	0.52	9.08	30.38	-1.85 (**)
gga-miR-1782	chr2	18.64	9.60	1.23	8.04	7.21	0.26	36.84	85.20	-1.31 (**)
gga-miR-144-5p	chr19	124.10	176.87	-0.24	69.58	33.80	1.14 (**)	828.62	540.38	0.51
gga-miR-22-3p	chr19	823.77	1269.17	-0.35	367.62	161.94	1.28 (**)	119.06	106.05	0.06
gga-miR-451	chr19	223.43	295.63	-0.13	522.28	361.48	0.63	315.00	124.52	1.23 (**)
gga-miR-199-5p	chr17, chr8	186.15	109.49	1.04 (**)	656.76	715.41	-0.02	1206.63	1040.83	0.11
gga-miR-1625-5p	chr15	44.92	109.58	-1.02 (**)	29.71	29.72	0.10	52.86	19.66	1.32 (**)
gga-let-7b	chr1	9.80	35.96	-1.61 (**)	1777.39	1585.62	0.26	123.33	189.46	-0.72
gga-miR-1664-5p	chr1	3.11	2.86	0.39	5.86	5.51	0.19	12.28	36.34	-1.67 (**)
gga-miR-6606-5p	chr1	757.50	1.85	8.95 (**)	489.02	203.69	1.36 (**)	113.19	0.00	7.77 (**)
novel_748	chr1	0.55	0.17	2.0	0.25	0.20	0.39	2.67	19.07	-2.94(**)

The highly differentially expressed miRNAs (| $\log_2$ (foldchange) | > 1; q-value < 0.01) in the comparisons were remarked as ** in the brackets.
